# Diversification of the Balloon bushcrickets (Orthoptera, Hexacentrinae, *Aerotegmina*) in the East African mountains

**DOI:** 10.1038/s41598-021-89364-4

**Published:** 2021-05-10

**Authors:** Beata Grzywacz, Elżbieta Warchałowska-Śliwa, Maciej Kociński, Klaus-Gerhard Heller, Claudia Hemp

**Affiliations:** 1 Institute of Systematics and Evolution of Animals, Polish Academy of Sciences, Sławkowska 17, 31-016 Kraków, Poland; 2 Unaffiliated, Magdeburg, Germany; 3 Department Plant Systematics, University of Bayreuth, Bayreuth, Germany

**Keywords:** Molecular biology, Zoology

## Abstract

East African mountains constitute a network of isolated habitat islands among dry savannah and are thus ideal for studying species diversification processes. This study elucidated the phylogenetic and phylogeographic relationships of all bushcricket species comprising the genus *Aerotegmina*. Our analysis indicated that large-scale climatic and topographic processes in Africa are likely to have driven speciation in this group, and revealed the cytogenetic traits of the species. Molecular phylogeny supported the monophyly of *Aerotegmina* and showed that the genus probably originated in the old Eastern Arc Mountains of Tanzania and Kenya. Two lineages were distinguished: small- and large-sized species with geographically distinct habitats. The underlying processes are thought to be eight dispersals, ten vicariance events, and one extinction event linked to repeated fragmentation of the African rainforest. Those processes, in conjunction with habitat change, probably also led to the spatial separation of the species into a northern clade with a diploid number of chromosomes 2n = 32 + X0 or 2n = 30 + neo-XY and a southern clade with a reduced number of chromosomes (2n = 28 + X0 or 24 + neo-X_1_X_2_Y). Karyotype analysis suggests that *Aerotegmina* is currently in the process of speciation.

## Introduction

Tropical montane rainforests are hotspots of species richness and offer diverse habitats for many endemic taxa^1,2^. Such high diversity may be the result of past climate changes and geological events inducing continual increases and decreases in forest areas^3,4^. However, tropical forest biodiversity is under great pressure from human activity and is vanishing rapidly worldwide^5^, also in East Africa^6,7^. The Eastern Arc Mountains in Kenya and Tanzania constitute one of the 17 most threatened hotspots in the world, which are crucial for the conservation of biodiversity and evolutionary heritage^2,8–10^. The Eastern Arc Mountains are a chain of isolated, geologically ancient mountains thought to have been covered by rainforests for millions of years^11,12^. The knowledge of African Orthoptera from that region is far from complete, particularly in terms of taxonomic placement and delimitation across all taxonomic levels. Many of these Orthoptera are flightless, indicating that geological and climatic events could have played a role in the evolution of this group. It has been suggested that the climate changes of the past one to three million years^13^ enhanced speciation in East Africa, as seen in various genera with arrays of morphologically and molecularly closely related species, e.g., in the subtribe Karniellina (subfamily: Conocephalinae^14–16^), the genus *Peronura* (subfamily: Phaneropterinae^17^), the genus *Parepistaurus* (subfamily: Coptacridinae^18^), and the family Lentulidae^19,20^. Most inland mountains, such as Mt. Kilimanjaro, adjacent to the geologically old northern branch of the Eastern Arc Mountains, can serve as time markers since their geological age is known. Species endemic to those young volcanoes enable the estimation of speciation processes in closely related species arrays of orthopteran genera present in young and old mountain ranges. The Meru and Kilimanjaro massifs are relatively young with an estimated age of two to three million years^21^, whereas the Eastern Arc Mountains are thought to be over 30 million years old^11^. Importantly, despite the relatively young age of the volcanoes, they harbor a high proportion of endemic orthopteran species, which implies rapid speciation in that region^22^.

The Balloon Bushcricket genus *Aerotegmina* is a perfect taxon for studying the role of climate and topography in determining patterns of species diversity and distribution. *Aerotegmina* belongs to the subfamily Hexacentrinae within the family Tettigoniidae and comprises five species so far^23^. The genus was described by Hemp^24^, with strongly inflated wings used for acoustical communication being its distinctive feature. Bioacoustics of this genus has been extensively studied, the call of some species being among the loudest known in insects^25^. Two groups of *Aerotegmina* species can be distinguished by their small or large body size as well as their extremely divergent song pattern^25^. According to Hemp^26^, *Aerotegmina* probably originated in the Eastern Arc Mountains. All of its species are canopy dwellers found in lowland to montane forests along the coast, in both old and young East African mountains. To date, only two *Aerotegmina* species (*A. kilimandjarica* and *A. shengenae*) have been studied cytogenetically, with the male diploid chromosome number being 2n = 32 + X0 in *A. kilimandjarica* and 2n = 26 + X0 in *A. shengenae*^27^.

At present, the molecular phylogeny of *Aerotegmina* is poorly known. The relationship of the genus was proposed by Hemp et al.^27^ based on morphological, bioacoustical, and chromosomal evidence. In this study, we provide comprehensive information on the molecular phylogeny and cytogenetics of this genus and discuss the ecological processes leading to species diversification and the current biogeography of the group. First, the taxonomic status of *Aerotegmina* species is assessed using genetic samples from across the range of the genus. Second, the molecular clock approach is used to investigate whether the timing of divergences within this clade corresponds with specific geological and climatic events in Africa. Phylogeographic tools were used to determine if speciation was affected by dispersal, vicariance, and extinction events. Finally, the cytogenetic characters of each species are provided.

## Material and methods

### Taxon sampling

**Figure 1:**
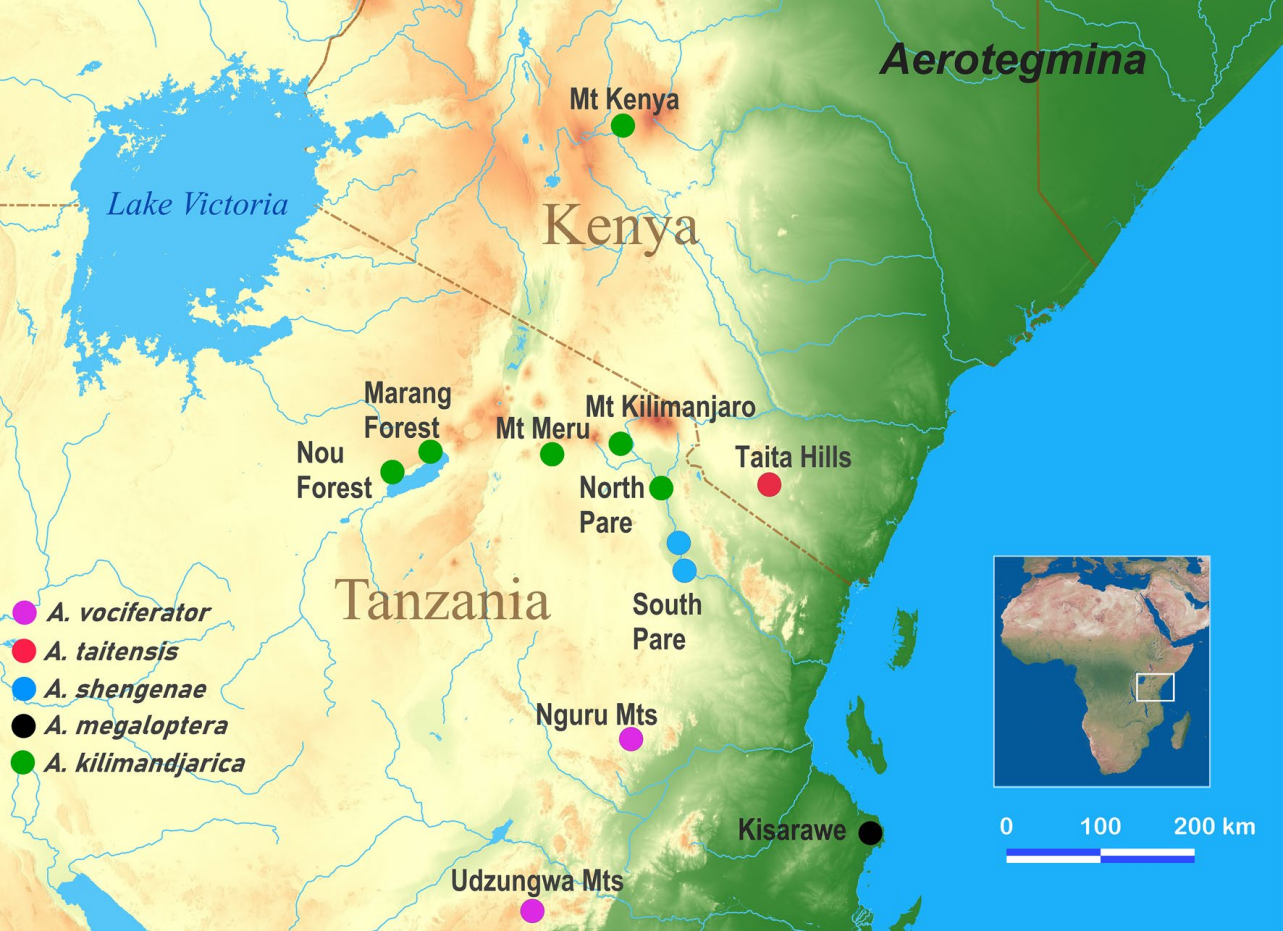
Map of collecting localities of analyzed specimens of *Aerotegmina*. Created by B. Grzywacz using QGIS 3.6 ‘Noosa’ (http://qgis.osgeo.org)^82^.

A total of 64 individuals from five *Aerotegmina* species, three individuals from two Hexacentrinae taxa (two individuals—*Hexacentrus* sp. and one—*Nepheliphila raptor* Hugel, 2010) and two individuals from two Meconematinae taxa (*Breviphisis* sp. and *Longiphisis* sp.) were collected in the field (Table S1, Fig. 1). Six outgroup Meconematinae taxa were selected for this study.

### DNA extraction, amplification, and sequencing

DNA was extracted from the legs of specimens using the NucleoSpin tissue kit (Macherey–Nagel, Germany) following the manufacturer’s protocol. Three molecular markers were used for reconstructing the interspecific relationships of *Aerotegmina*: cytochrome c oxidase subunit I (COI), 16S ribosomal RNA (16S), and histone 3 (H3), which have proved useful in previous studies of closely related groups of East African bushcrickets (e.g.,^14–16,28,29^). PCR amplification and cycle sequencing of the three gene fragments were carried out with the following primers: LCO and HCO for COI^30^, 16a and 16b for 16S^31^, and H3fwd and H3rev for H3^32^. The amplification reaction was performed in a 20 µL reaction volume consisting of 2.0 µL of 10 × PCR buffer, 25 mM of MgCl_2_, 10 mM of dNTP mixture, 10 µM of forward and reverse primers, 1–3 µL of genomic DNA, and 5U/µL of Gold Taq DNA polymerase (Syngen, Poland), and sterile deionized water. To amplify COI, the following PCR protocol was used: 36 cycles at 94 °C for 1 min, at 48 °C for 1 min, and at 72 °C for 2 min, with a final extension at 72 °C for 7 min. The PCR conditions for the 16S gene were as follows: 38 cycles at 94 °C for 45 s, at 48 °C for 45 s, and at 72 °C for 80 s, with a final extension at 72 °C for 1 min. In the case of H3, the PCR procedure consisted of 30 cycles at 94 °C for 15 s, at 55 °C for 15 s, and 72 °C for 30 s, with a final extension at 72 °C for 1 min. All PCR products were purified using Exo-BAP Mix (EURx, Poland, following the standard protocol) and were sequenced using the BrilliantDye v3.1 Terminator Cycle Sequencing Kit (NimaGen, Netherlands), and ABI 3730XL sequencer. The sequences generated for this study were deposited in the GenBank database under the accession numbers given in Table S1. The sequences were checked and edited using CodonCode Aligner (CodonCode Corporation). The DNA sequences of *Amytta kilimandjarica*, *A. meruensis*, *A. merumontana*, and *A. olindo* were selected from GenBank to be included as an outgroup in the dataset. For all genes, separate alignments were conducted using ClustalX^33^. Sequences were checked for protein-coding frame shifts to detect pseudogenes using MEGA X^34^ and compared with sequences from GenBank through a Blast search. The potential saturation of the nucleotide substitution was checked for all markers separately and for two separate partitions of COI (with codon positions 1 + 2 and codon position 3). Saturation was examined through the substitution saturation test^35^ implemented in DAMBE^36^. The partition homogeneity test^37^ in PAUP^38^ with 1000 replicates was used to determine whether all regions (COI, 16S, H3) could be combined in a unique data matrix.

### Phylogenetic analyses

Two methods were used to establish phylogenetic relationships: Bayesian inference (BI) and maximum likelihood (ML). A model of sequence evolution was selected with MrModeltest software^39^. MrBayes^40^ was used to obtain a BI tree. The symmetrical gamma distribution model (SYM + G) was the best-fit for the COI, 16S and H3 considered separately. Posterior probabilities were based on two independent Markov chain Monte Carlo (MCMC) runs, each composed of four chains (three heated and one cold). Markov chains were run for 6 million generations with sampling every 100 generations. The default 25% burn-in was applied before constructing the majority-rule consensus tree. The convergence of analyses was validated by evaluating likelihood values using Tracer^41^. ML estimates of phylogeny were implemented in IQ-TREE^42^.

### Divergence time estimation and biogeographic analysis

To estimate the divergence time of each clade without any molecular clock constraint, a Bayesian approach with MCMC integration was used to date the most recent common ancestor using Beast software^43^. The analysis was run for 20 million generations with sampling every 1000 generations and a 10% burn-in. A Yule branching process with a lognormal relaxed clock for estimating the posterior probability density of divergence time was implemented. Convergence to the stationary distribution and the effective sample size of the model parameters were checked using Tracer. Maximum clade credibility trees were generated with TreeAnnotator^43^. The tree was calibrated using two calibration points from Mugleston et al.^44^: an *Aerotegmina*–*Hexacentrus* split 37.5 Mya and *Aerotegmina–*Meconematinae split 90 Mya, with a normal distribution centered on 37.5 Mya and 90 Mya, respectively, at one standard deviation to reflect the uncertainty associated with the original estimate. Additionally, for the two on Mts Kilimanjaro and Meru endemic meconematine sister pairs *Amytta kilimandjarica* and *A. merumontana* and *A. olindo* and *A. meruensis* the molecular clock was calibrated using the event of volcanism representing the origin of Mt. Kilimanjaro with a normal distribution centered on 1.75 Mya^29^.

The maximum clade credibility tree and distribution file were uploaded to show the biogeographic reconstruction obtained by statistical dispersal-vicariance analysis (S-DIVA^45^) implemented in RASP^46^. The number of maximum ancestral areas was set to 4, whereas trees and a condensed tree were generated by Beast. S-DIVA was conducted with the default settings. Eleven biogeographical regions related to the sampled sites of *Aerotegmina* were defined: Mt. Kilimanjaro, Marang Forest, Mt. Kenya, Mt. Meru, Nou Forest, Taita Hills, North Pare Mountains, South Pare Mountains, Kazimzumbwi Forest Reserve (Kisarawe), Nguru Mountains, and Udzungwa Mountains.

### Cytotaxonomic analyses

The study material consisted of *Aerotegmina kilimandjarica* (new data, three males: HE6, HE9, HE43 and seven described in^27^: CH6825, CH6827, CH6850-54), *A. shengenae* (new data, five males: CH8676, CH8677, CH8317, CH8318, CH8320, HE127, HE128, HE131 and one described in^27^: CH7268), *A. taitensis* (three males: HE119, HE120, HE122), *A. vociferator* (one male: HE123), and *A. megaloptera* (five males: HE39, HE40, HE43, CH8026, CH8309). Chromosome preparations were obtained from gonads and hepatic caeca. Testes and somatic body parts were incubated in a hypotonic solution (0.9% sodium citrate), fixed in ethanol:acetic acid (3:1) and stored at 2 °C until use. The fixed material was squashed in 45% acetic acid. Coverslips were removed by the dry ice procedure and then the preparations were air-dried. Constitutive heterochromatin was revealed by the C-banding technique as described by Sumner^47^. Chromosomes were examined using a Nikon Eclipse 400 microscope with a CCD DS-U1 camera and NIS-Elements BR2, and the images were processed and arranged with Adobe Photoshop. For each individual, at least three spermatogonial mitotic metaphases and 15 meiotic divisions in males (except *A. vociferator*) were examined.

To estimate the ancestral character states for the number of chromosomes we used a continuous-time Markov chain model implemented in "phytools"^48^ R package^49^. 500 trees were simulated to obtain state probability. It was visualized on the modified Beast chronogram.

## Results

### Phylogeny, dating, and biogeography

Samples were successfully sequenced for all markers. A total of 1500 positions were aligned within the dataset. The results of the substitution test for each gene alignment are summarized in Table 1. Calculated *P*-values were significant for all gene alignments and Iss (index of substitution saturation) values were lower than Iss.c (critical index of substitution saturation) in all cases. No saturation of the phylogenetic signal was observed for the COI, 16S and H3 dataset.

**Table 1:** Results of the substitution saturation tests performed in DAMBE.

Dataset	ISS	ISS.c S	*P*	ISS.c A	*P*
COI (1 + 2)	0.0969	0.6953	0	0.3978	0
COI (3)	0.3802	0.6368	0	0.3601	0.4996
16S	0.5401	0.7312	0.0001	0.4912	0
H3	0.0777	0.7204	0	0.5955	0

**Figure 2:**
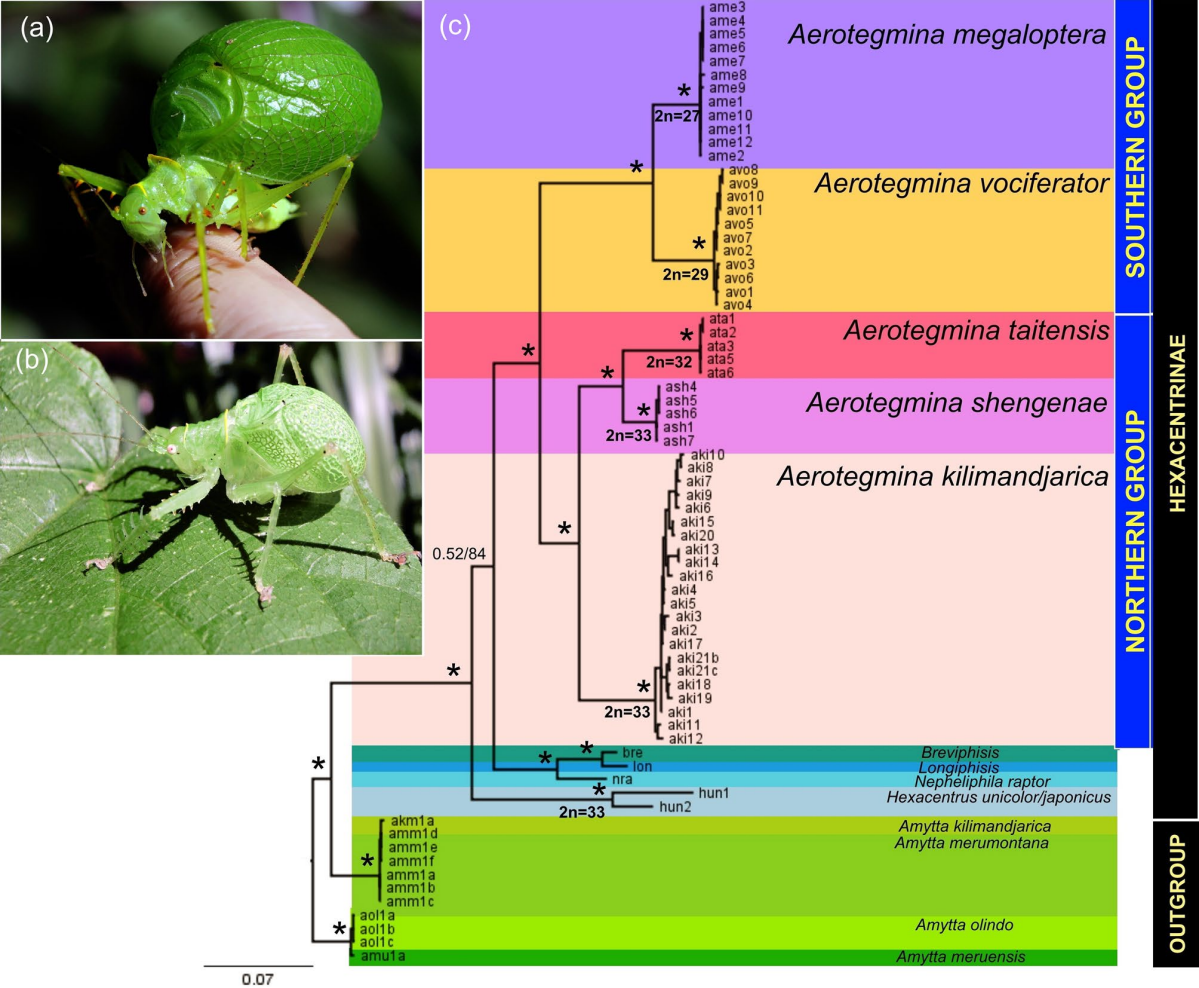
*Aerotegmina megaloptera* (**a**), *A. shengenae* (**b**), photo C. Hemp. Bayesian inference tree from a dataset including COI, 16S, and H3 sequences of Hexacentrinae genera (**c**). Bayesian (BI) and Maximum likelihood (ML) topologies were consistent, so only one tree is shown. Values at nodes correspond to posterior probability/bootstrap support; *designates ≤ 0.99 and 95% values for BI and ML, respectively. Diploid chromosome numbers for *Aerotegmina* species are plotted on the phylogenetic tree. Scale bar: number of substitutions per nucleotide position. OUT—outgroup, HEXA—Hexacentrinae.

The topologies obtained from BI and ML analyses for the combined dataset were similar. Figure 2 shows the BI consensus tree based on combined COI, 16S, and H3 data. All specimens of each *Aerotegmina* species formed a monophyletic group. The *Aerotegmina* species were split into two major lineages showing morphological differences. The northern group consisted of the three small-sized taxa: the widespread *A. kilimandjarica* being the sister group to the two endemic species *A. shengenae* from the South Pare Mountains in Tanzania and *A. taitensis* from the Taita Hills in Kenya. The southern group consisted of two large-sized species: *A. vociferator* from the Nguru and Udzungwa populations clustered together and *A. megaloptera* from coastal Tanzania. Small molecular differences at the population level could be detected between *A. kilimandjarica* specimens from Mt. Kilimanjaro and the North Pare Mountains, despite this they formed one subclade within the northern group.

**Figure 3:**
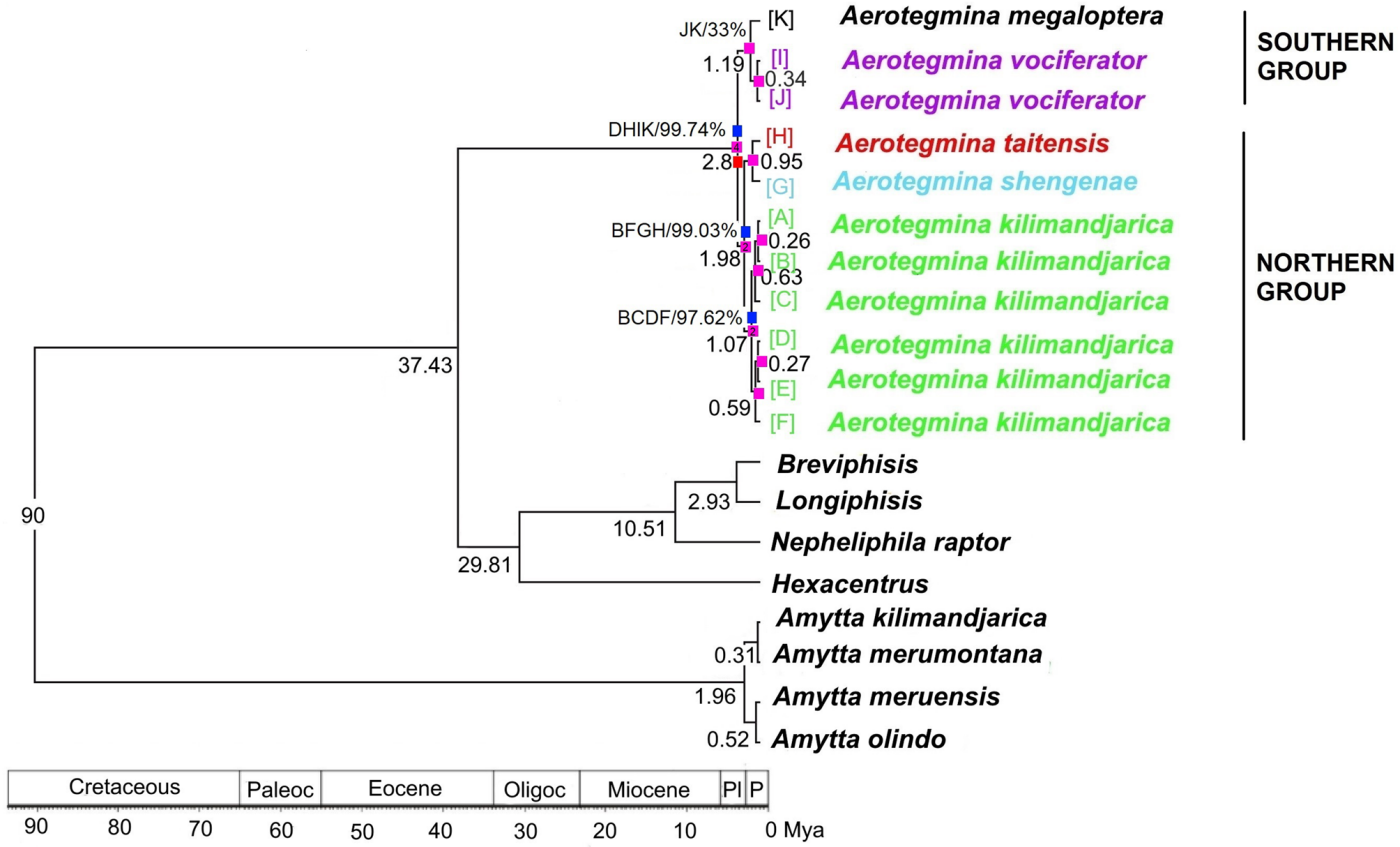
Reconstructed geographic ranges and dated phylogeny of *Aerotegmina* obtained through phylogenetic analysis using Beast software with a relaxed molecular clock model (Beast tree is given). The values indicated under the branches represent the mean ages of lineage divergence; acronyms on the nodes and at the tip labels indicate geographic areas: [A]—North Pare Mountains, [B]—Marang Forest, [C]—Mt. Kenya, [D]—Mt. Meru, [E]—Nou Forest, [F]—Mt. Kilimanjaro, [G]—South Pare Mountains, [H]—Taita Hills, [I]—Udzungwa Mountains, [J]—Nguru Mountains, [K]—Kazimzumbwi Forest Reserve (Kisarawe); the values above branches support the predicted distribution. The different colour squares on the branches close to the nodes represent different events: pink—vicariance, navy blue—dispersal and red—extinction; digit inside the square means how many events were at the node (showing only values greater than 1). Mya—million years ago, Paleoc—Paleocene, Oligoc—Oligocene, Pl—Pliocene and P—Pleistocene.

The topology resulting from the molecular clock was similar to the Bayesian tree (Fig. 3). Based on the molecular clock analysis and a priori calibration, the divergence of the small- and large-sized species was dated at *ca.* 2.8 Mya (1.81–4.04 Mya, 95% confidence interval). The major clades of the small-sized species are likely to have diverged *ca.* 1.98 Mya (1.16–2.89 Mya, 95% confidence interval) and those of the large-sized species *ca.* 1.19 Mya (0.19–2.24 Mya, 95% confidence interval). Divergence events among the two main species lineages were placed in the late Pliocene (*ca.* 5.33–2.6 Mya).

The biogeographic scenario indicates that the current distribution pattern of *Aerotegmina* results from eight dispersals, ten vicariance, and one extinction events (Fig. 3). The main divergence of *Aerotegmina* was dated to the late Pliocene (*ca* 2.8 Mya). The S-Diva analysis on the Beast tree identified Kazimzumbwi Forest Reserve (Kisarawe), Mt. Meru, the Taita Hills and the Udzungwa Mountains, after four dispersals, one extinction and one vicariance as ancestral area for *Aerotegmina* species. For the southern group (large-sized species: *A. megaloptera, A. vociferator*) of *Aerotegmina* the ancestral areas were Kazimzumbwi Forest Reserve (Kisarawe), the Udzungwa Mountains and the Nguru Mountains after one vicariance. Finally, *A. megaloptera* occupied Kazimzumbwi Forest Reserve (Kisarawe) while *A. vociferator* occurred in the Udzungwa Mountains and the Nguru Mountains*.* For the northern group (small-sized species: *A. shengenae, A. taitensis, A. kilimandjarica*) of *Aerotegmina* the ancestral areas were the South Pare, the Taita Hills, Mt. Kilimanjaro and Marang Forest after a vicariance and two dispersals. *Aerotegmina shengenae* dispersed from the ancestral area to the South Pare Mountains whereas *A. taitensis* to the Taita Hills. The S-DIVA analysis for the *A. kilimandjarica* species group found one vicariance event and two dispersals, leading to the current distribution pattern of this species, today occurring in montane forests of the Manyara Escarpment (Nou Forest, Marang Forest), on Mt. Kilimanjaro and on Mt. Kenya.

### Chromosomes

A comparison of the karyotypes of five *Aerotegmina* species revealed differences in the number of chromosomes (2n), the chromosome morphology (including X and Y chromosomes), the fundamental number of chromosome arms (FN), the sex chromosome system, and C-banding. The examined *Aerotegmina* males had 33 to 27 chromosomes and one of three sex determination systems: X0, neo-XY, or neo-X_1_X_2_Y.

**Figure 4:**
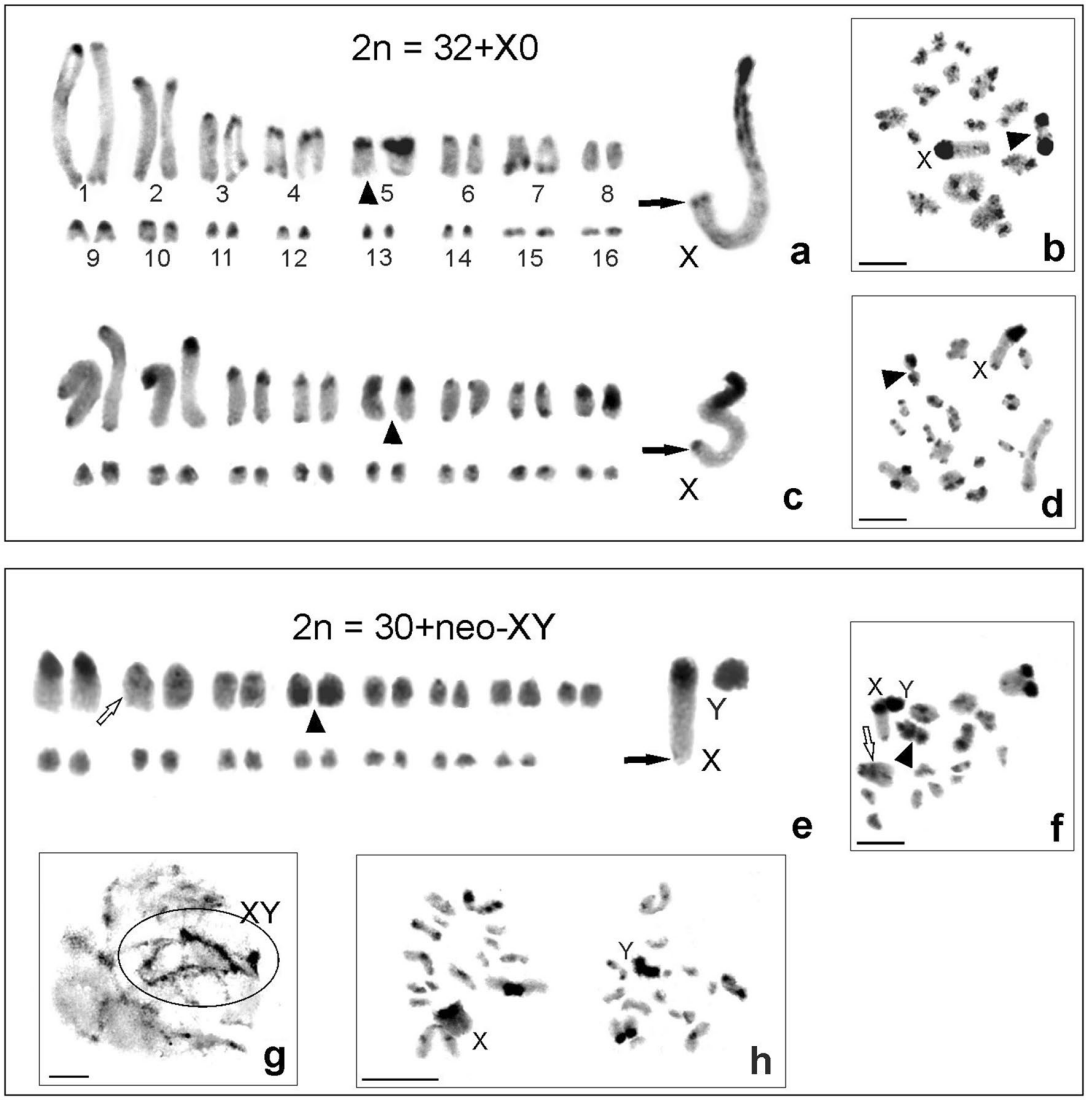
Heterochromatin C-banding at karyograms arranged from mitotic metaphase (**a**, **c, e**), pachytene (**g**), diakinesis/metaphase I (**b**, **d**, **f**), and metaphase II (**h**) for male chromosome complements in *Aerotegmina kilimandjarica* (**a**, **b**) and *A. shengenae* (**c**, **d**) 2n = 32 + X0 and *A. taitensis* 2n = 30 + neo-XY (**e**–**h**). X chromosome with thick paracentromeric and telomeric C-bands (black arrows) (**a**, **c, e**); medium-sized pairs exhibit thick paracentromeric heterochromatin (arrowheads) (**a**–**f**); marked by white arrow weak interstitial C-band in second pair (**e**, **f**). In the neo-XY system, sex chromosomes form a ring (**g**), both acrocentric neo-X and neo-Y are connected by telomeric segments (**f**); metaphase II with 16 chromosomes (15 + neo-X and 15 + neo-Y) (**h**). Scale bar = 10 µm.

The standard karyotypes of *Aerotegmina kilimandjarica* and *A. shengenae* (both new data) were characterized by a chromosome number of 2n = 32 + X0, FN = 33 in males. The chromosomes were acrocentric, consisting of one long and 15 medium-sized or short pairs gradually decreasing in size, as well as an X chromosome, the largest element in the set. In both species, one medium-sized autosome pair (probably the fifth) was heterochromatinized, demonstrating in some individuals megameric characteristics. In the X chromosome, thick paracentromeric C-bands and thin telomeric ones were found in both species. Thin or thick paracentromeric and thin telomeric C-positive heterochromatin blocks occurred in most autosomes in *A. kilimandjarica* (new data and^27^), whereas in *A. shengenae* the second pair had thick C-bands (Fig. 4a–d). At diakinesis/metaphase I cells with this chromosome/bivalent number were clearly visible (Fig. 4b, d, respectively).

*A. taitensis* males had a chromosome number of 2n = 30 + neo-XY, FN = 32. Two long and thirteen medium-sized/short autosome pairs, a neo-X and neo-Y chromosome were acrocentric. This species had thin paracentromeric C-bands in most autosomes, except for the first and third pairs with a thick C-heterochromatin block. Additionally, a very weak interstitial C-band occurred in the second pair and a thick distal C-block in the fourth pair. The neo-X was the largest chromosome in the set, with thick paracentromeric and minute telomeric C-positive blocks, whereas the medium-sized neo-Y chromosome was strongly heterochromatinized (Fig. 4e, f, h). During pachytene, the sex chromosomes formed a ring or a loop (Fig. 4g), whereas in diakinesis/metaphase I the neo-X and neo-Y chromosomes were joined near both distal parts (Fig. 4f). At metaphase II there were 16 chromosomes, including a neo-X or neo-Y (Fig. 4h).

**Figure 5:**
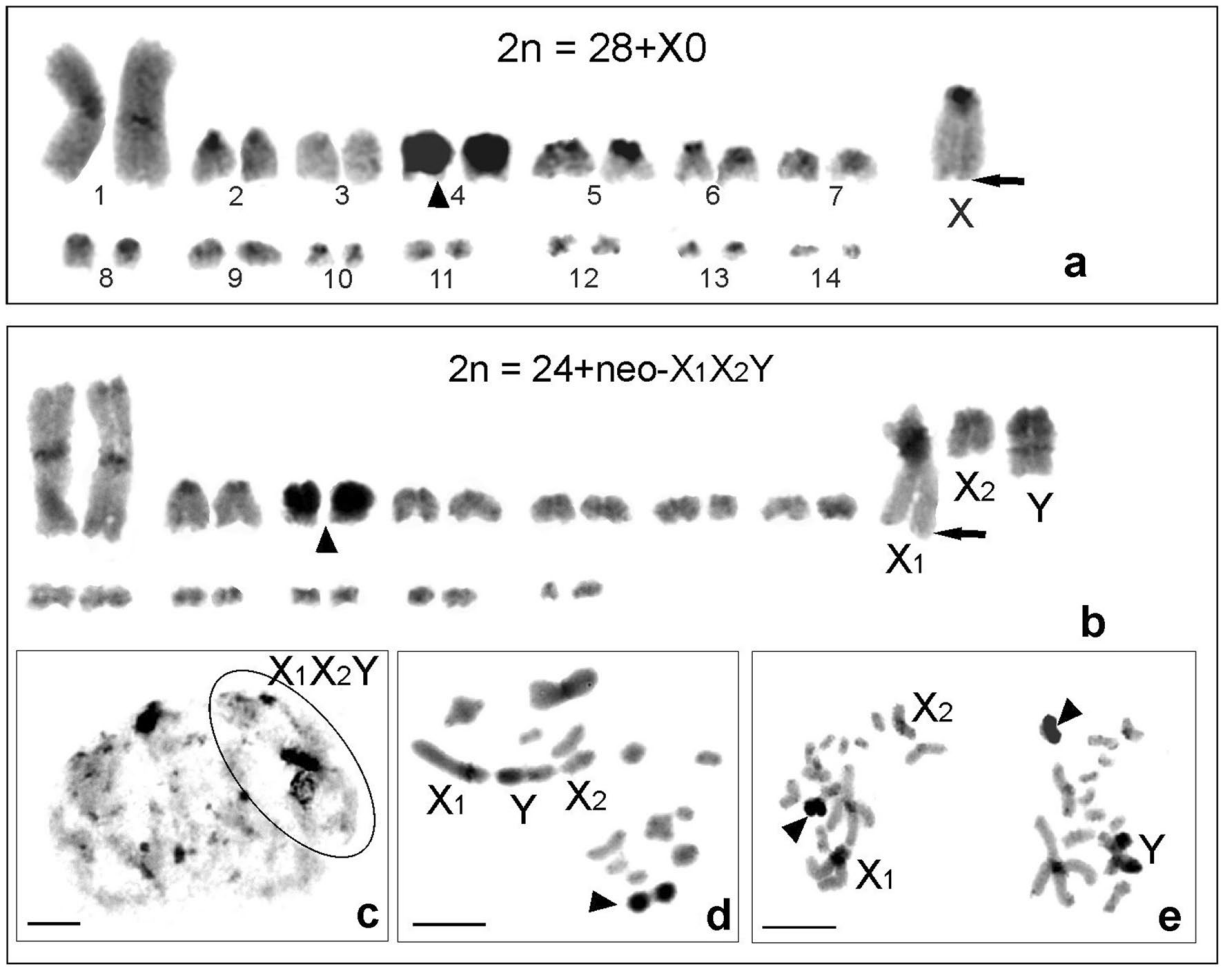
C-banded karyograms arranged from mitotic metaphases of *Aerotegmina vociferator* 2n = 28 + X0 (**a**) and *A. megaloptera* 2n = 24 + neo-X_1_X_2_Y (**b**–**e**); medium-sized megameric pair (arrowheads) (**a, b, d, e**). The neo-X_1_X_2_Y system: pachytene with positively heteropycnotic X_1_ (**c**), terminally associated sex trivalent (**d**), and metaphase II with two types of secondary spermatocytes with 14 chromosomes (12 + neo-X_1_ and neo-X_2_) and 13 chromosomes (12 + neo-Y) (**e**). Scale bar = 10 µm.

In *A. vociferator* (a single male with somatic mitosis), the chromosome number was reduced to 2n = 28 + X0, FN = 31. Fourteen pairs of autosomes, one large metacentric and thirteen medium-sized/short acrocentric, were arranged into two groups; thin C-bands were located in the paracentromeric region in most autosomes, except for the third or fourth pair (megameric) with strong thick C-bands occupying most of the chromosomes. The X chromosome was the second element of the set with thick paracentromeric and minute telomeric C heterochromatin bands (Fig. 5a).

The lowest chromosome number, 2n = 24 + neo-X_1_X_2_Y, FN = 31 was found in *A. megaloptera*. This species had one large metacentric and eleven acrocentric autosome pairs. Heterochromatin formed thin C-positive paracentromeric bands in most of the chromosomes; the medium-sized (probably third) chromosome pair could be considered megameric. A submetacentric neo-X_1_ was the second element in the karyotype, whereas an acrocentric neo-X_2_ was similar in length to the medium-sized autosomes. The submetacentric neo-Y was about two times as large as the neo-X_2_ (Fig. 5b). During the early prophase (zygotene-pachytene-diplotene), the neo-X_1_ was positively heteropycnotic (Fig. 5c). At diakinesis, the sex chromosomes were generally connected by a single terminal chiasma or end-to-end association. During metaphase I, the neo-X_1_ was terminally associated with the “left” arm of the metacentric Y, whereas the acrocentric neo-X_2_ was associated with the “right” arm of the neo-Y (Fig. 5d). After anaphase I, two types of metaphase II complements were formed, with 14 chromosomes (12 + neo-X_1_, neo-X_2_) and 13 chromosomes (12 + neo-Y), respectively (Fig. 5e). Thick paracentromeric C-bands were found on both bi-armed neo-X_1_ and neo-Y, whereas a thin C-positive band was observed in the neo-X_2_ sex chromosome. Additionally, a minute telomeric C-block was observed in the neo-X_1_ (Fig. 5b).

**Figure 6:**
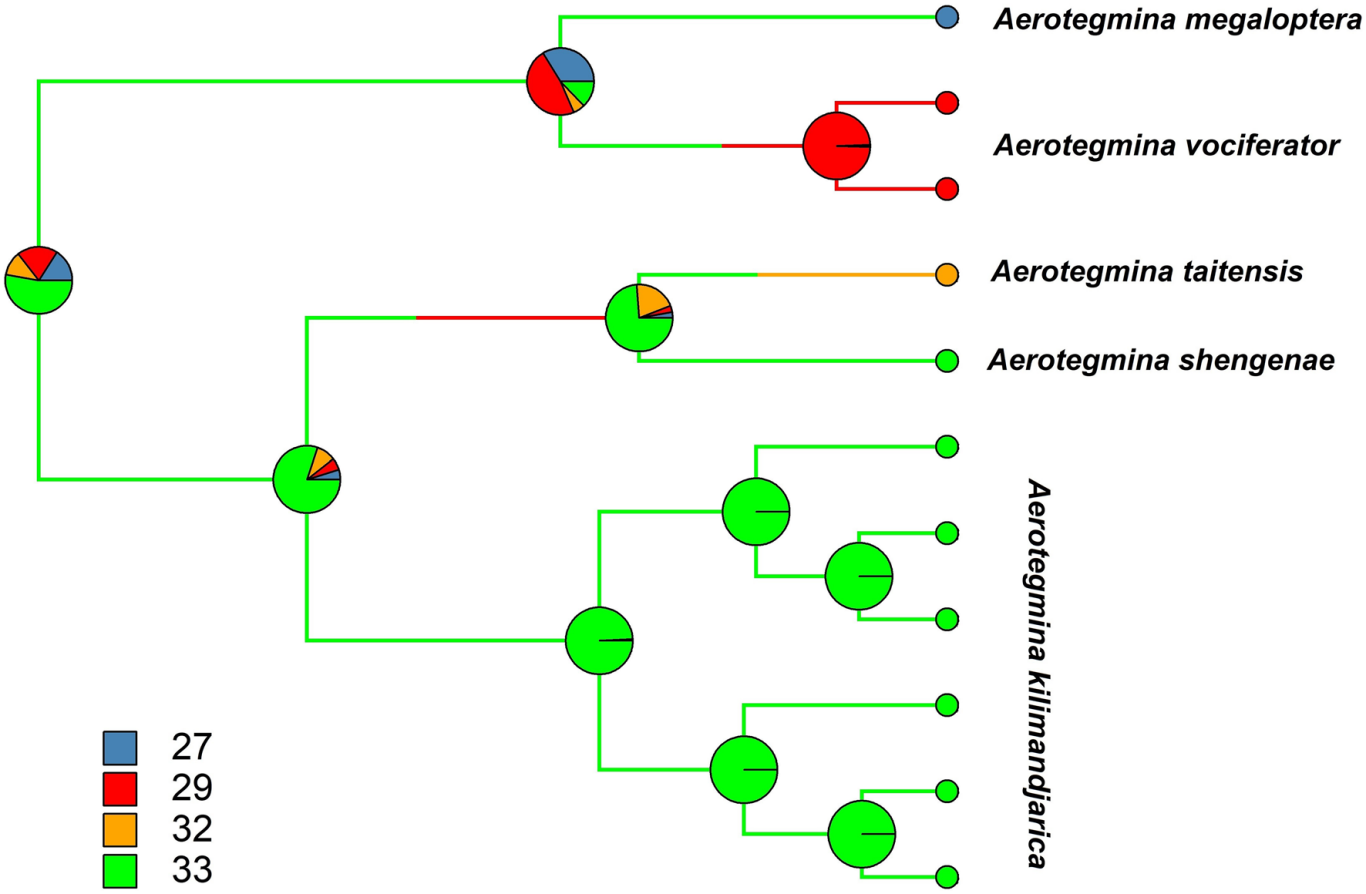
The ancestral character states for number of chromosomes mapped on the modified *Aerotegmina* Beast chronogram. The different colour branches present different chromosome numbers (explained by squares in the left corner). Circles represent the posterior probability of being in each state across all the edges and nodes of the tree.

The ancestral character states analysis (Fig. 6) revealed the ancestor of *Aerotegmina* most likely had a chromosome number 2n = 33 chromosomes.

## Discussion

The presented molecular analyses confirmed the monophyly of the genus *Aerotegmina* and a species diversification timeline was established*.* Speciation coincided with the climatic and geological phenomena leading to dispersal, which resulted in a clear spatial phylogenetic pattern. Moreover, phylogeny was found to be associated with differences in body size and in the number and structure of chromosomes.

### Two morphological groups of *Aerotegmina*

This study is the first comprehensive molecular analysis of *Aerotegmina*. All previous works were based on morphological, bioacoustical, or cytogenetic data^25,27^. The present findings showed that *Aerotegmina* is monophyletic within Hexacentrinae (posterior probability support, PP = 1.0; bootstrap support, BP = 100) and is a sister clade to *Breviphisis*, *Longiphisis*, and *Nepheliphila* (Fig. 2). The morphology of the species of northern Tanzania and Kenya (*A. kilimandjarica, A. shengenae, A. taitensis*) and southern Tanzania (*A. megloptera* and *A. vociferator*) showed differences, especially in body size. The northern group consisted of two morphologically very closely related species, *A. shengenae* and *A. taitensis,* endemic to the South Pare Mountains and the Taita Hills, respectively, belonging to the ancient Eastern Arc Mountains. A similar pattern of geographically separated species is also seen in various other genera of flightless Orthoptera, e.g., *Peronura*^50^, *Rhainopomma*^19,51^, *Parepistaurus*^52^, and *Melanoscirtes*^53^. Specimens from different *A. kilimandjarica* populations formed a sister group to the sister pair of *A. taitensis* and *A. shengenae*. These results agree with previous preliminary molecular studies^54^. The present data do not confirm the assumption that *A. shengenae* is the most basal species of the group, as suggested in previous studies^27,55^ based on morphology (small, incompletely closed acoustic chamber) and acoustics (less loud than *A. kilimandjarica*). The southern group, formed by the large-sized species *A. megaloptera* and *A. vociferator* represented a sister group to all small-sized species of northern Tanzania and southern Kenya, which is consistent with the morphological differences between these two groups.

Our findings stand in opposition to the current taxonomic status of the genera *Breviphisis* and *Longiphisis*^56^. Probably, the two genera should be transferred from Meconematinae to the subfamily Hexacentrinae, in contrast to the concept of Gorochov^57^ but further studies are required to confirm their taxonomic position.

### Fragmentation of forest cover boosting speciation of *Aerotegmina*

The aridification-induced fragmentation of the forest cover isolated the ancestors of *Aerotegmina* probably in the old forests of the Eastern Arc range during the Eocene. With the onset of the East African aridification (33–20 Mya), the forests of the Eastern Arc Mountains were isolated, which would explain the high endemicity of the area (e.g.,^8,58–60^). In their studies of the family Annonaceae, Couvreur et al.^61^ found that all East African taxa diversified prior to the Pleistocene and that their origins coincide with known periods of aridification and geological activity in Africa which repeatedly isolated the Guinea-Congolian rainforest from the East African one. A major split in Hexacentrinae on the genus level was observed in the Eocene at 37.43 Mya. The results of S-DIVA revealed that *Aerotegmina* most recent common ancestor (2.8 Mya) was already present in a wide range covering the Taita Hills, Kazimzumbwi Forest Reserve (Kisarawe), Mt. Meru and the Udzungwa Mountains but not the South Pare Mountains as previously suggested^27^. It is difficult to infer the biogeographic histories of *Aerotegmina* but probably the old Eastern Arc Mountains of Tanzania and Kenya were the habitat of the ancestors of this genus. Interesting is that the Taita Hills and the South Pare Mountains share a pair of closely related endemic species (*A. shengenae* and *A. taitensis*) but it is unclear which species is the basal one*.* The split of the small-sized *Aerotegmina* species from the large-sized species occurred in the Pliocene around 2.8 Mya. It overlapped with a global climatic shift which resulted in a major increase of grassland habitats and consequently a change in species composition^62,63^. Humid periods reconnecting forest cover triggered a dispersal event leading to the separation of the northern small-sized species and the large-sized species distributed further south which changed their habitat during their evolution. All small-sized *Aerotegmina* species are restricted to montane forests in the Eastern Arc Mountains (*A. shengenae*, *A. taitensis, A. kilimandjarica*) while a habitat change is obvious for the large-sized species: *A. vociferator* now occurs in montane to submontane forests in the Udzungwa and Nguru Mountains, while *A. megaloptera* is found in lowland wet forests. Also the speciation of Lentulidae (Orthoptera) coincides with the fragmentation of the African rainforests, and subsequently the East African lentulids diversified rapidly in the Eastern Arc Mountains along with the aridification of large parts of Africa and strong geological activity^19,20^.

It was believed that *A. kilimandjarica* was endemic to Mt. Kilimanjaro because it is an immobile flightless dweller of the canopy layer of submontane and montane forest^24^. However, more intensive sampling revealed that *A. kilimandjarica* is the only species widespread on both young and old mountains in the area, being distributed from the North Pare Mountains, across Mt. Kilimanjaro, Mt. Meru, and the Ngorongoro area to central Kenya (Mt. Sabuk, Mt. Kenya). In this study mainly specimens from the North Pare Mountains, Mt. Kilimanjaro, and only single individuals from Mt. Kenya, Mt. Meru and the Nou and Marang Forest Reserves on the Manyara Escarpment were analyzed. Small molecular differences were detected between the investigated populations of these areas, which is in line with Schultz^54^, suggesting present isolation of these populations from each other and a recent dispersal of this species in East Africa^27^. A specimen from the Marang Forest Reserve, adjacent to the Nou Forest Reserve, clustered with the North Pare and Kilimanjaro individuals. The current genetic differences could result from fragmentation of a formerly widespread species. Climatic fluctuations could affect the connectivity among *A. kilimandjarica* populations both increasing and preventing migration of individuals. Migration events occurred during humid and cooler times when the montane forest zones were reconnected. It is assumed that migration was not regulated by accidental shifts, e.g. wind or birds. More analyses on the faster evolving genes should be undertaken to clarify the more recent spread of *A. kilimandjarica*. It is likely that *A. kilimandjarica* evolved in the geologically old Eastern Arc Mountains and spread from there once Mt. Kilimanjaro was formed about 2 Mya, opening montane forests corridors further west (Mt. Meru, Ngorongoro area) and north (up to Mt. Kenya). Further analyses of additional populations of *A. kilimandjarica* would elucidate whether the ice age cycles creating montane forest corridors triggered a dispersal of that species and would enable a deeper understanding of the complex relationships between a changing climate, forests and *A. kilimandjarica* occurrence.

### The evolutionary history of *Aerotegmina* under a cytogenetic perspective

The use of molecular phylogenetics to analyse the karyotype evolution is an objective tool to determine the direction of changes that cause chromosome variation^64,65^. A comparison of the chromosomes of the five studied *Aerotegmina* species (including previous data^27^) revealed differences between their karyotypes. The chromosomal complement was very variable in both the diploid number in males and the sex determination system (2n = 32 + X0, 2n = 30 + neo-XY, 2n = 28 + X0, 2n = 24 + neo-X_1_X_2_Y). The basal chromosome number may be directly related to the chromosomal variability of *Aerotegmina* and its two molecularly and morphologically distinguished groups. In the northern group, the ancestral chromosome number and sex determination mechanism, 2n = 32 + X0 (FN = 33) found in *A. kilimandjarica*^27^ and *A. shengenae* was reduced to 2n = 30 + neo-XY (FN = 32) in *A. taitensis.* In the case of *A. shengenae* a different chromosome number was reported in a previous paper: 2n = 26 + X0^27^ compared to 2n = 32 + X0 found in the present study. However, since the previous findings were based on a single male only, a mistake cannot be ruled out and it is unclear whether the reported data reflect intraspecific variability or an incorrectly tagged individual. The 33 chromosome set in males is thus probably a plesiomorphic character for both *A. kilimandjarica* and *A. shengenae*, since a similar chromosome number is also found in *Hexacentrus*^26^. The neo-XY sex chromosome system is derived from the X0 system and is rarely observed in tettigoniids. However, two East African *Spalacomimus* (Hetrodinae) species have revealed divergent origins of this system in studies involving both classical and molecular cytogenetic techniques^66^. Based on C-banding analysis, one may suggest that the neo-XY system arose in *A. taitensis* (endemic to the Taita Hills in Kenya) due to rearrangements resulting from a tandem fusion between the original acrocentric X chromosome and part of the medium-sized chromosome pair with thick (double) C-bands near the distal end. The acrocentric neo-Y (a medium-sized member of the set), is partially homologous to the autosome, with a double C-band. In Hexacentrinae, a neo-XY has been found in the Indian species *Euhexacentrus annulicornis*^67^, but in that case the karyotype had only metacentric chromosomes, including both neo-X and neo-Y originated by multiple rearrangements.

In the southern group two species *A. vociferator* and *A. megaloptera* showed diminished chromosome numbers with 2n = 28 + X0 (FN = 31) and 2n = 24 + neo-X_1_X_2_Y (FN = 31), respectively. In both species, one Robertsonian (centric) fusion between large/medium-sized autosomes affected the first pair of autosomes (bi-armed chromosomes). The neo-X_1_X_2_Y system is very rare in tettigoniids^68,69^. The series of rearrangements giving rise to this multiple sex chromosome system derived directly from the X0 system, and probably occurred in *A. vociferator* by translocations/and or inversion between the X chromosome and two pairs of autosomes. To confirm such a mechanism of chromosomal rearrangements in this species it would be necessary to apply markers obtained by silver impregnation and fluorescence in situ hybridization using ribosomal DNA and telomeric DNA.

It should be noted, that upon C-staining, chromosomal regions showed some quantitative and qualitative variation between the analyzed species in terms of constitutive heterochromatin. Characteristics of *Aerotegmina* species common to the northern and the southern groups were: i) heterochromatin blocks in the paracentromeric region with a thick C-band and a minute telomeric C-band in the X and/or neo-X chromosomes, and, ii) one medium-sized/small pair was heterochromatinized. This set of similarities in heterochromatin was exclusively found in this monophyletic group of Hexacentrinae.

The role of chromosomal rearrangements (CRs), including the reduced chromosome numbers in the evolution and in originating reproductive barriers has been investigated having a causative role in isolating species or populations in some genera of Hemiptera^70,71^, Hymenoptera: Formicidae^72^, Diptera^73^, Coleoptera^74,75^, Lepidoptera^76^, and Orthoptera^77,78^. To better understand the role of chromosome changes in species diversification interaction between chromosomal and genetic variability requires analysis in the context of species’ geographical distribution (e.g.^65,70,77,79,80^).

Considering the current contrasting geographical range and respective karyotypes of *Aerotegmina* species, the fixation of different CRs either by genetic drift or isolation could have been triggered by recurrent climatic events that continuously changed the environment. The split into two groups would have involved probably dispersion, vicariance (allopatry) and extinction events, geographic speciation via vicariance has been the most documented to date and is a common speciation model across tropical Africa^81^. The geographic isolation of the ancestor of *Aerotegmina* species due to humid and arid periods (see above) changing their habitat led to a split of the two lineages within the genus (northern small-sized species and southern large-sized species). Chromosomal changes seem to be a consequence of adaptation to different habitats, but mutations (including chromosomal changes) always take place before adaptation. Chromosomal rearrangements could be more or less adaptive depending on affected genes/regions and their interactions. In any case, they represent a very important reproductive barrier themselves and, further, a possible mechanism of speciation.

When mapped on the phylogenetic tree, the observed cytogenetic characteristics reflect the phylogenetic pattern, suggesting that certain cytogenetic character stages, like a lowered number of chromosomes, occur in closely related species. Genetic differentiation and habitat specialization was accompanied by chromosomal variation in *Aerotegmina*. Additional studies are required focusing on the cytogenetic analysis of more individuals especially of *A. vociferator* and populations/localities of other species to increase the cytogenetic data on *Aerotegmina*.

## Conclusions

We found that *Aerotegmina* species evolved as a result of vicariance, dispersal and extinction events resulting in a clear pattern of body size variation and spatial separation of clades. These events were probably linked to geological processes and past climate changes. Habitat fragmentation within the Eastern Arc Mountain could have boosted allopatric speciation through vicariance in a previously isolated mountain region. Our results support the idea of a montane origin of the genus *Aerotegmina* dated in the Eocene, although the majority of species-level diversification appears to have taken place from the Pliocene onwards. However, the structure of karyotypes and varying chromosome numbers indicate that *Aerotegmina* speciation is still an ongoing process. Various processes and mechanisms of speciation are interconnected and are responsible for the patterns of genetic diversity that can be observed at different population and phylogenetic levels in nature.

## Supplementary Information

Supplementary Information 1.
