# Supplementary material for: Diversification of the Balloon bushcrickets (Orthoptera, Hexacentrinae, Aerotegmina) in the East African mountains
Source: Sci Rep. 2021 May 10;11:9878. doi: 10.1038/s41598-021-89364-4 (PMC8110521; doi:10.1038/s41598-021-89364-4)
Supplement: Supplementary file 1 — Supplementary Information 1. [file 41598_2021_89364_MOESM1_ESM.pdf]

Diversification of the Balloon Bushcrickets (Orthoptera, Hexacentrinae, *Aerotegmina*) in the East African mountains

Beata Grzywacz[1*], Elżbieta Warchałowska-Śliwa[1], Maciej Kociński[1], Klaus-Gerhard Heller[2], Claudia Hemp[3]

[1] Institute of Systematics and Evolution of Animals, Polish Academy of Sciences, Sławkowska 17, 31-016 Kraków, Poland
[2] Independent researcher
[3] University of Bayreuth, Dept. Plants Systematics, Bayreuth, Germany

[*]Corresponding author: Beata Grzywacz
Institute of Systematics and Evolution of Animals, Polish Academy of Sciences, Sławkowska 17, 31-016 Krakow, Poland, e-mail: grzywacz@isez.pan.krakow.pl

Table S1. Locality data for the specimens sequenced and specimens used for cytogenetics

Species	Locality	Chromosome	Isolate	GenBank Accession Numbers		
				COI	16S	H3
***Aerotegmina kilimandjarica* Hemp, 2001**	Tanzania: Mt. Kilimanjaro	-	aki1	MT787583	MT775526	MT784670
***Aerotegmina kilimandjarica* Hemp, 2001**	Tanzania: Mt. Kilimanjaro	-	aki2	MT787594	MT775527	MT784671
***Aerotegmina kilimandjarica* Hemp, 2001**	Tanzania: Mt. Kilimanjaro	HE6	aki3	MT787598	MT775528	MT784672
***Aerotegmina kilimandjarica* Hemp, 2001**	Tanzania: Mt. Kilimanjaro	-	aki4	MT787599	MT775529	MT784673
***Aerotegmina kilimandjarica* Hemp, 2001**	Tanzania: North Pare	-	aki5	MT787600	MT775530	MT784674
***Aerotegmina kilimandjarica* Hemp, 2001**	Tanzania: North Pare	-	aki6	MT787601	MT775531	MT784675
***Aerotegmina kilimandjarica* Hemp, 2001**	Tanzania: North Pare	HE9	aki7	MT787602	MT775532	MT784676
***Aerotegmina kilimandjarica* Hemp, 2001**	Tanzania: North Pare	-	aki8	MT787603	MT775533	MT784677
***Aerotegmina kilimandjarica* Hemp, 2001**	Tanzania: North Pare	-	aki9	MT787604	MT775534	MT784678
***Aerotegmina kilimandjarica* Hemp, 2001**	Tanzania: North Pare	-	aki10	MT787584	MT775535	MT784679

***Aerotegmina kilimandjarica* Hemp, 2001**	Tanzania: North Pare	CH8675f	aki11	MT787585	MT775536	MT784680
***Aerotegmina kilimandjarica* Hemp, 2001**	Tanzania: North Pare	CH8675f	aki12	MT787586	MT775537	MT784681
***Aerotegmina kilimandjarica* Hemp, 2001**	Tanzania: North Pare	-	aki13	MT787587	MT775538	MT784682
***Aerotegmina kilimandjarica* Hemp, 2001**	Tanzania: Mt. Kilimanjaro	HE43	aki14	MT787588	MT775539	MT784683
***Aerotegmina kilimandjarica* Hemp, 2001**	Tanzania: Mt. Kilimanjaro	-	aki15	MT787589	MT775540	MT784684
***Aerotegmina kilimandjarica* Hemp, 2001**	Tanzania: Mt. Kilimanjaro	-	aki16	MT787590	MT775541	MT784685
***Aerotegmina kilimandjarica* Hemp, 2001**	Tanzania: Mt. Kilimanjaro	-	aki17	MT787591	MT775542	MT784686
***Aerotegmina kilimandjarica* Hemp, 2001**	Tanzania: Mt. Meru	-	aki18	MT787592	MT775543	MT784687
***Aerotegmina kilimandjarica* Hemp, 2001**	Tanzania: Nou Forest	-	aki19	MT787593	MT775544	MT784688
***Aerotegmina kilimandjarica* Hemp, 2001**	Tanzania: Marang Forest	-	aki20	MT787595	MT775545	MT784689
***Aerotegmina kilimandjarica* Hemp, 2001**	Kenya: Mt. Kenya	-	aki21b	MT787596	MT775546	MT784690
***Aerotegmina kilimandjarica* Hemp, 2001**	Kenya: Mt. Kenya	-	aki21c	MT787597	MT775547	MT784691
***Aerotegmina megaloptera* Hemp, 2013**	Tanzania: Kazimzumbwi Forest Reserve (Kisarawe)	CH8309	-	-	-	-
***Aerotegmina megaloptera* Hemp, 2013**	Tanzania: Kazimzumbwi Forest Reserve (Kisarawe)	-	ame1	MT787605	MT791271	MT784692
***Aerotegmina megaloptera* Hemp, 2013**	Tanzania: Kazimzumbwi Forest Reserve (Kisarawe)	CH8026	ame2	MT787609	MT791272	MT784693
***Aerotegmina megaloptera* Hemp, 2013**	Tanzania: Kazimzumbwi Forest Reserve (Kisarawe)	-	ame3	MT787610	MT791273	MT784694
***Aerotegmina megaloptera* Hemp, 2013**	Tanzania: Kazimzumbwi Forest Reserve (Kisarawe)	-	ame4	MT787611	MT791274	MT784695
***Aerotegmina megaloptera* Hemp, 2013**	Tanzania: Kazimzumbwi Forest Reserve (Kisarawe)	-	ame5	MT787612	MT791275	MT784696
***Aerotegmina megaloptera* Hemp, 2013**	Tanzania: Kazimzumbwi Forest Reserve (Kisarawe)	-	ame6	MT787613	MT791276	MT784697
***Aerotegmina megaloptera* Hemp, 2013**	Tanzania: Kazimzumbwi Forest Reserve (Kisarawe)	-	ame7	MT787614	MT791277	MT784698
***Aerotegmina megaloptera* Hemp, 2013**	Tanzania: Kazimzumbwi Forest Reserve (Kisarawe)	-	ame8	MT787615	MT791278	MT784699
***Aerotegmina megaloptera* Hemp, 2013**	Tanzania: Kazimzumbwi Forest Reserve (Kisarawe)	-	ame9	MT787616	MT791279	MT784700
***Aerotegmina megaloptera* Hemp, 2013**	Tanzania: Kazimzumbwi Forest Reserve (Kisarawe)	HE40	ame10	MT787606	MT791280	MT784701
***Aerotegmina megaloptera* Hemp, 2013**	Tanzania: Kazimzumbwi Forest Reserve (Kisarawe)	HE39	ame11	MT787607	MT791281	MT784702
***Aerotegmina megaloptera* Hemp, 2013**	Tanzania: Kazimzumbwi Forest Reserve (Kisarawe)	HE43	ame12	MT787608	MT791282	MT784703
***Aerotegmina shengenae* Hemp, 2006**	Tanzania: South Pare	CH8676	-	-	-	-
***Aerotegmina shengenae* Hemp, 2006**	Tanzania: South Pare	CH8677	-	-	-	-
***Aerotegmina shengenae* Hemp, 2006**	Tanzania: South Pare	CH8317	-	-	-	-
***Aerotegmina shengenae* Hemp, 2006**	Tanzania: South Pare	CH8318	-	-	-	-
***Aerotegmina shengenae* Hemp, 2006**	Tanzania: South Pare	CH8320	-	-	-	-

Aerotegmina shengenae **Hemp, 2006**	Tanzania: South Pare	-	ash1	MT787617	MT791253	MT784704
Aerotegmina shengenae **Hemp, 2006**	Tanzania: South Pare	-	ash2	MT787618	MT791254	MT784705
Aerotegmina shengenae **Hemp, 2006**	Tanzania: South Pare	-	ash3	MT787619	MT791255	MT784706
Aerotegmina shengenae **Hemp, 2006**	Tanzania: South Pare	-	ash4	MT787620	MT791256	MT784707
Aerotegmina shengenae **Hemp, 2006**	Tanzania: South Pare	-	ash5	MT787621	MT791257	MT784708
Aerotegmina shengenae **Hemp, 2006**	Tanzania: South Pare	-	ash6	MT787622	MT791258	MT784709
Aerotegmina shengenae **Hemp, 2006**	Tanzania: South Pare	-	ash7	MT787623	MT791259	MT784710
Aerotegmina taitensis **Hemp, 2013**	Kenya: Taita Hills	-	ata1	MT787624	MT791307	MT784711
Aerotegmina taitensis **Hemp, 2013**	Kenya: Taita Hills	HE119	ata2	MT787625	MT791308	MT784712
Aerotegmina taitensis **Hemp, 2013**	Kenya: Taita Hills	HE120	ata3	MT787626	MT791309	MT784713
Aerotegmina taitensis **Hemp, 2013**	Kenya: Taita Hills	HE121	-	-	-	-
Aerotegmina taitensis **Hemp, 2013**	Kenya: Taita Hills	-	ata5	MT787627	MT791310	MT784714
Aerotegmina taitensis **Hemp, 2013**	Kenya: Taita Hills	-	ata6	MT787628	MT791311	MT784715
Aerotegmina vociferator **Hemp, 2019**	Tanzania: Udzungwa Mountains	-	avo1	MT787629	MT791260	MT784716
Aerotegmina vociferator **Hemp, 2019**	Tanzania: Nguru Mountains	-	avo2	MT787632	MT791261	MT784717
Aerotegmina vociferator **Hemp, 2019**	Tanzania: Udzungwa Mountains	-	avo3	MT787633	MT791262	MT784718
Aerotegmina vociferator **Hemp, 2019**	Tanzania: Udzungwa Mountains	-	avo4	MT787634	MT791263	MT784719
Aerotegmina vociferator **Hemp, 2019**	Tanzania: Nguru Mountains	-	avo5	MT787635	MT791264	MT784720
Aerotegmina vociferator **Hemp, 2019**	Tanzania: Udzungwa Mountains	-	avo6	MT787636	MT791265	MT784721
Aerotegmina vociferator **Hemp, 2019**	Tanzania: Nguru Mountains	-	avo7	MT787637	MT791266	MT784722
Aerotegmina vociferator **Hemp, 2019**	Tanzania: Nguru Mountains	HE123	avo8	MT787638	MT791267	MT784723
Aerotegmina vociferator **Hemp, 2019**	Tanzania: Nguru Mountains	-	avo9	MT787639	MT791268	MT784724
Aerotegmina vociferator **Hemp, 2019**	Tanzania: Nguru Mountains	-	avo10	MT787630	MT791269	MT784725
Aerotegmina vociferator **Hemp, 2019**	Tanzania: Nguru Mountains	-	avo11	MT787631	MT791270	MT784726
Breviphisis **sp**	Madagascar	-	bre	MT787640	MT791283	MT784727
Hexacentrus **sp**	Japan: Okinawa	-	hun1	MT787641	MT791251	MT784728
Hexacentrus **sp**	Japan: Okinawa	-	hun2	MT787642	MT791252	MT784729
Longiphisis **sp**	Madagascar	-	lon	MT787643	MT791284	MT784730
Nepheliphila raptor **Hugel, 2010**	Mauritius	-	nra	MT787644	MT791285	-
Amytta kilimandjarica **Hemp, 2001**	Tanzania: Mt. Kilimanjaro	-	akm1a	MF383080	MF383007	-

***Amytta merumontana* Hemp, 2017**	Tanzania: Mt. Meru	-	amm1a	MF383081	MF383008	MF383048
***Amytta merumontana* Hemp, 2017**	Tanzania: Mt. Meru	-	amm1b	MF383082	MF383009	MF383049
***Amytta merumontana* Hemp, 2017**	Tanzania: Mt. Meru	-	amm1c	MF383083	MF383010	MF383050
***Amytta merumontana* Hemp, 2017**	Tanzania: Mt. Meru	-	amm1d	MF383054	MF383011	MF383051
***Amytta merumontana* Hemp, 2017**	Tanzania: Mt. Meru	-	amm1e	MF383085	MF383012	MF383052
***Amytta merumontana* Hemp, 2017**	Tanzania: Mt. Meru	-	amm1f	MF383086	MF383013	MF383053
***Amytta meruensis* Hemp, 2017**	Tanzania: Mt. Meru	-	amu1a	-	MF383019	-
***Amytta olindo* Hemp, 2001**	Tanzania: Mt. Kilimanjaro	-	aol1a	MF383092	MF383020	MF383054
***Amytta olindo* Hemp, 2001**	Tanzania: Mt. Kilimanjaro	-	aol1b	MF383093	MF383021	MF383055
***Amytta olindo* Hemp, 2001**	Tanzania: Mt. Kilimanjaro	-	aol1c	MF383094	MF383022	MF383056
